# The influence of Percoll® density gradient centrifugation before cryopreservation on the quality of frozen wisent (*Bison bonasus*) epididymal spermatozoa

**DOI:** 10.1186/s12917-022-03408-z

**Published:** 2022-08-10

**Authors:** Maria Eberhardt, Sylwia Prochowska, Anna M. Duszewska, Ann Van Soom, Wanda Olech, Wojciech Niżański

**Affiliations:** 1 Department of Reproduction and Clinic of Farm Animals, Faculty of Veterinary Medicine, Wroclaw University of Environmental and Life Sciences, Plac Grunwaldzki 49, 50-366 Wrocław, Poland; 2 Department of Morphological Sciences, Faculty of Veterinary Medicine, Warsaw University of Life Sciences, Nowoursynowska 159 St, 02-787 Warsaw, Poland; 3 Department of Reproduction, Obstetrics and Herd Health, Faculty of Veterinary Medicine, University of Ghent, Salisburylaan 133, B-9820 Merelbeke, Belgium; 4 Department of Animal Genetics and Conservation, Institute of Animal Sciences, Warsaw University of Life Sciences, Ciszewskiego 8 St, 02-786 Warsaw, Poland

**Keywords:** Wisent, Spermatozoa, Percoll®, Freezability, Cryopreservation

## Abstract

**Background:**

The wisent (*Bison bonasus*) is a species that has undergone a population bottleneck. Homozygosity is prevalent within the population and may have a negative impact on semen quality in wisent bulls. Semen samples containing a large amount of functionally and morphologically impaired or dead spermatozoa have lower tolerance for cryopreservation process. Such samples are prone to involve damage acrosomes, to produce and release reactive oxygen which negatively affects proper function of spermatozoas. It is a good practice to select intact and viable gametes before subjecting the sample to cryopreservation to improve the efficiency of this process. The aim of this study was to assess the ability of Percoll® density gradient centrifugation in order to improve the quality of wisent spermatozoa after cryopreservation. Spermatozoa samples were analysed with computer-assisted semen analysis system and flow cytometry.

**Results:**

Percoll® density gradient centrifugation resulted in increased percentage of motile spermatozoa, higher proportion of spermatozoa with normal morphology and proper functionality but also in a significant reduction of the total number of gametes. Nevertheless, the concentration of frozen spermatoza was still sufficient for obtaining a few complete insemination doses suggested for cattle from each epididymis.

**Conclusions:**

While creating a high-quality genetic reserve, for in vitro fertilisation purposes, eliminating detritus and improving the overall quality of samples is more important than total number of spermatozoa. For these reasons, the achievement of higher post thaw quality of spermatozoa justifies the purification of samples by centrifugation in a Percoll® density gradient prior to the cryopreservation process.

## Background

The wisent (*Bison bonasus*, Linnaeus 1758) is the largest land mammal currently living in Europe [1]. Centuries of hunting, poaching, and armed conflicts led to the last free-ranging individual being found dead in 1919 in in the Białowieska Primeval Forest [1]. At the beginning of the twentieth century, conservationists initiated activities aimed to recover this species. The transfer of one male and two females from the zoological gardens to the enclosure in Białowieża is considered to be the beginning of the restitution programme in Poland [1]. Due to the effectiveness of breeding efforts, the current population is consistently increasing in size and the total worldwide number of individuals reached 9111 at the end of 2020 (data from The European Bison Pedigree Book as of December 31st) However, the wisent is a species that has undergone a population bottleneck which led to homozygosity [1–3]. An impoverished gene pool is an inevitable problem for all endangered populations [4]. For this reason, recovery programmes could be supported by Assisted Reproductive Techniques (ART), which enable saving genetic resources through mating least related individuals [4]. Important in those programmess is the cryopreservation of reproductive cells [5] and their preparation for this process which is crucial for obtaining a good quality genetic reserve [6].

It is well known that the freezing process cause modifications in structure of male gametes [6] and those changes are not fully reversible after thawing [7]. Ice formation, chemical toxicity, and overproduction of reactive oxygen species (ROS) is causing damage to sperm membranes. In comparison to fresh semen, frozen/thawed spermatozoa are predisposed to undergo morphological changes like acrosome abnormalities and are characterised by lower motility, reduced mitochondrial potential, increased plasma membrane permeability and lipid peroxidation [7]. These damages can reduce fertility of post-thawed spermatozoa [8]. However, the basic factor limiting the effectiveness of cryopreservation process is the initial quality of the obtained spermatozoa [9]. Samples containing large number of functionally and morphologically impaired and dead spermatozoa display a lower tolerance of the cryopreservation process than those characterized by good initial quality [10]. Impaired cells release acrosome contents and ROS that negatively affect properly functioning gametes [11]. Additional components which have a negative impact on properly functioning spermatozoa are blood formed elements, epithelial cells, or fragments of epididymal tissue. Removal of this debris may have a beneficial effect on the cryopreserved gametes [11, 12]. Therefore, it is good practice to select normal spermatozoa before subjecting the sample to cryopreservation in order to improve the efficiency of this process [10].

In present wisent population, homozygous effects occur which results in numerous abnormalities in sperm morphology and low sperm quality. In the current wisent population, homozygous effects occur, resulting in numerous abnormalities in sperm morphology and low overall sperm quality [13]. Therefore, the initial pre-freezing sperm selection to improve the efficiency of cryopreservation process was taken into consideration.

There are multiple semen purification techniques available [6, 14–16]. Percoll® density gradient centrifugation is a method based on sperm cell density that can be correlated to spermatozoal stage of maturation and integrity. Male gamets with a normal nucleus are denser and are deposited in the area of greater density. Furthermore, motile spermatozoa deposit faster than cells with impaired motility due to the alignment of movements with the centrifugal force used [6]. This colloidal solution of silica coated with polyvinylpyrrolidone (PVP) has been successfully used in bull, ram, boar, and human semen samples for removing undesirable spermatozoa from ejaculates [6, 16].

Wisent reproduction has been discussed in several articles [17–25]. However, there is a paucity of information focused on detailed characteristic of the male gamete in context of cryopreservation process in this species [26]. According to our best knowledge, no studies have been conducted using Percoll® density gradient centrifugation to select motile wisent spermatozoa and, thus, improve the quality of frozen samples.

The aim of our study was to compare the efficiency of the cryopreservation process of wisent epididymal spermatozoa in TRIS buffer-based egg yolk extender with and without previous Percoll® density gradient centrifugation by using computer-assisted semen analysis (CASA) and flow cytometry.

## Results

Percoll® density gradient centrifugation resulted in a significant reduction in total number of spermatozoa, with loses ranging from 43.87 to 86.67% (Tables 1 and 2).

**Table 1:** Total number of spermatozoa from separate epididymis (L and R represent a pair of epididymides from each individual from 1 to 4)

Epididymis	Initial number of spermatozoa [×10^6^]	Post centrifugation number of spermatozoa [×10^6^]	Losses (%)
1 L	410.00	129.50	68.41
1R	499.90	320.00	43.87
2 L	900.00	120.00	86.67
2R	860.00	232.50	72.97
3 L	960.00	520.00	45.83
3R	1960.00	720.00	63.27
4 L	1173.00	294.00	74.94
4R	85.00	441.00	48.42

**Table 2:** Number of spermatozoa obtained prior and after freezing- thawing (10^6^) from separate epididymis (L and R represent a pair of epididymides from each individual from 1 to 4)

	Fresh parameters						Post thaw parameters				
	TOTAL [×10^6^]		LIVE [×10^6^]		MOTILE [×10^6^]		TOTAL [×10^6^]		LIVE [×10^6^]		MOTILE [×10^6^]
	Control	Percoll	Control	Percoll	Control	Percoll	Control	Percoll	Control	Percoll	Control
Epididymis 1 L	728.00	410.00	607.88	342.35	364.00	225.50	728.00	129.50	83.72	53.10	14.56
Epididymis 1R	499.60	499.90	482.11	482.40	299.76	299.94	499.60	320.00	124.90	214.40	49.96
Epididymis 2 L	900.00	900.00	760.50	760.50	270.00	270.00	900.00	120.00	180.00	43.20	360.00
Epididymis 2R	900.00	860.00	722.70	690.58	360.00	344.00	900.00	232.50	342.00	156.94	360.00
Epididymis 3 L	600.00	960.00	474.00	758.40	360.00	576.00	600.00	520.00	306.00	301.60	240.00
Epididymis 3R	800.00	1960.00	632.00	1548.40	520.00	1274.00	800.00	720.00	288.00	518.40	160.00
Epididymis 4 L	160.00	1173.00	138.40	1014.65	96.00	703.80	160.00	294.00	60.80	97.20	56.00
Epididymis 4R	160.00	855.00	143.20	765.23	96.00	513.00	160.00	441.00	37.60	227.12	40.00

**Fig. 1:**
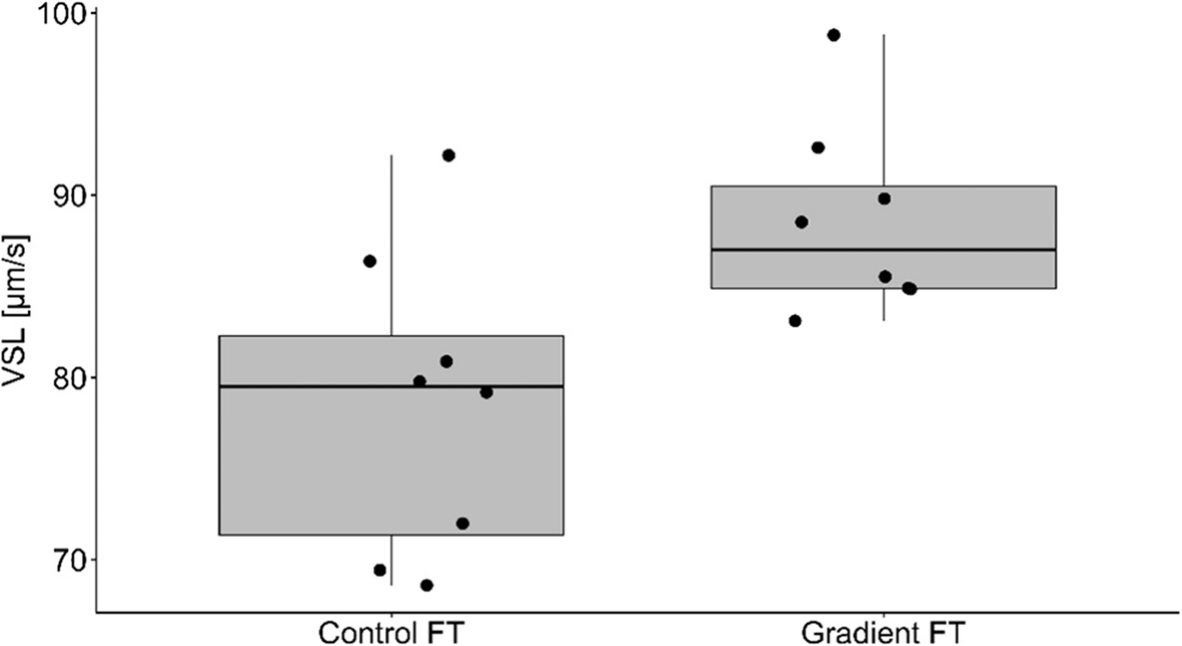
VSL- straight line velocity (*p* < 0.05)

**Fig. 2:**
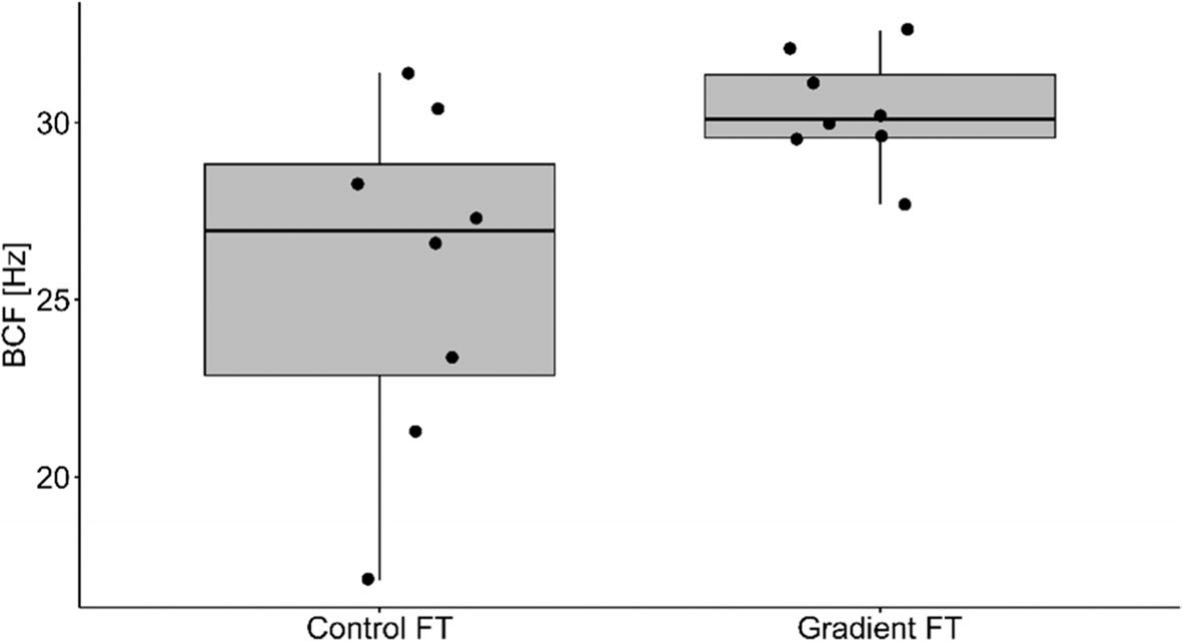
BCF- beat cross frequency (*p* < 0.05)

**Fig. 3:**
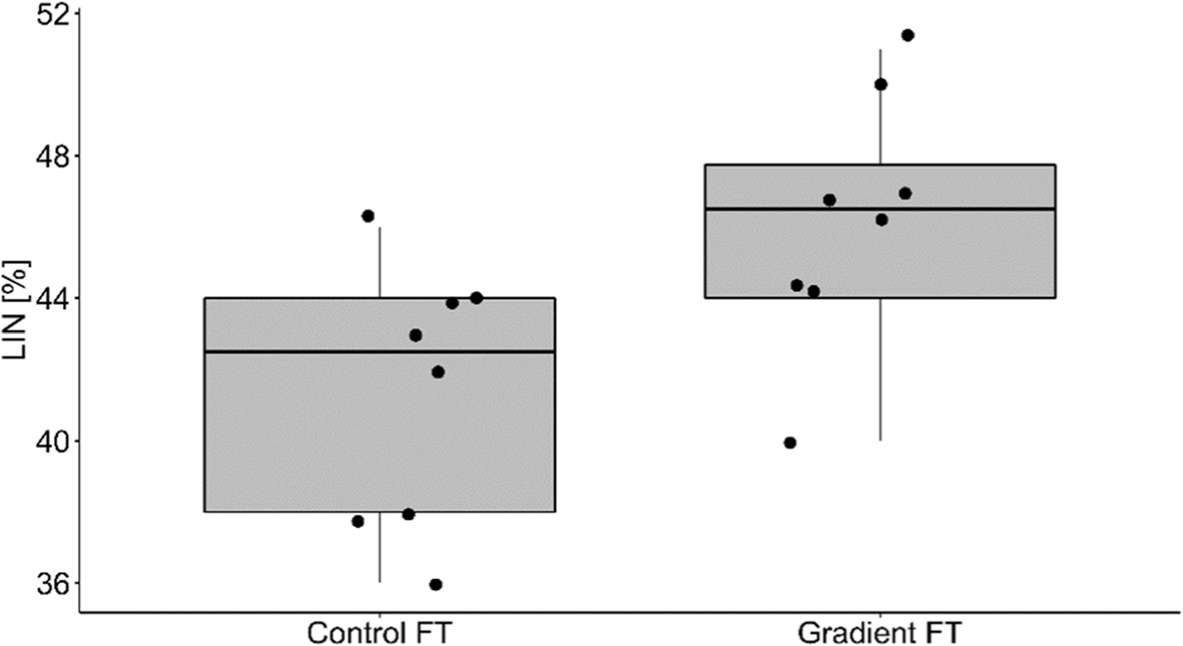
LIN- the linearity of spermatozoa movement (*p* < 0.05)

**Fig. 4:**
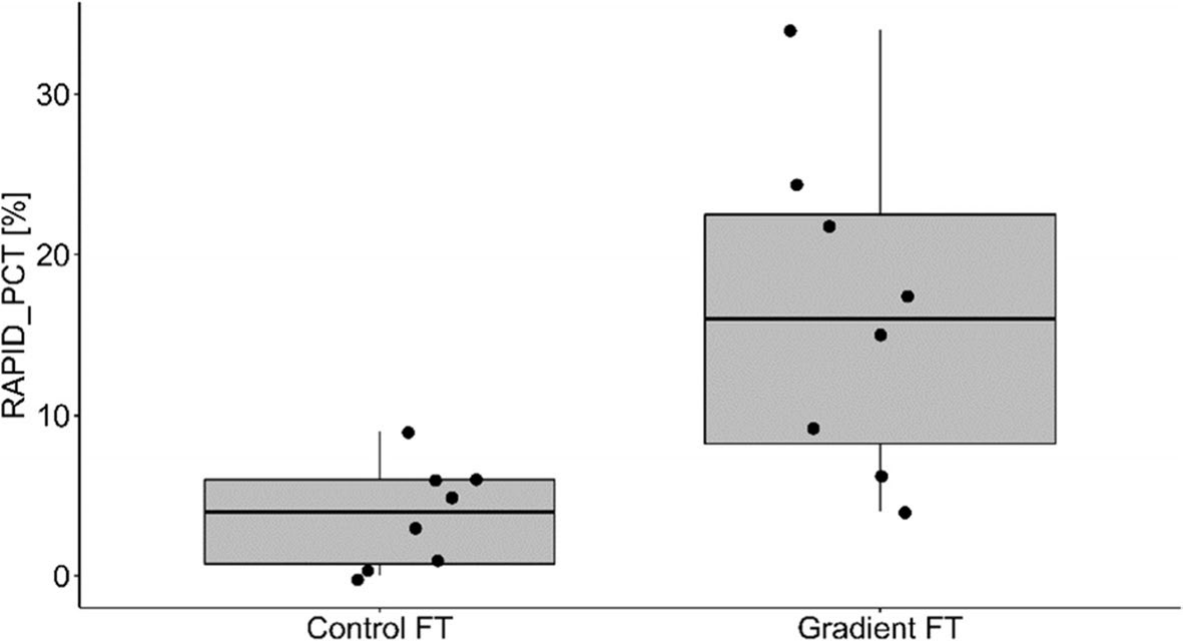
RAPID- subpopulations of spermatozoa showing rapid movement (*p* < 0.05)

**Fig. 5:**
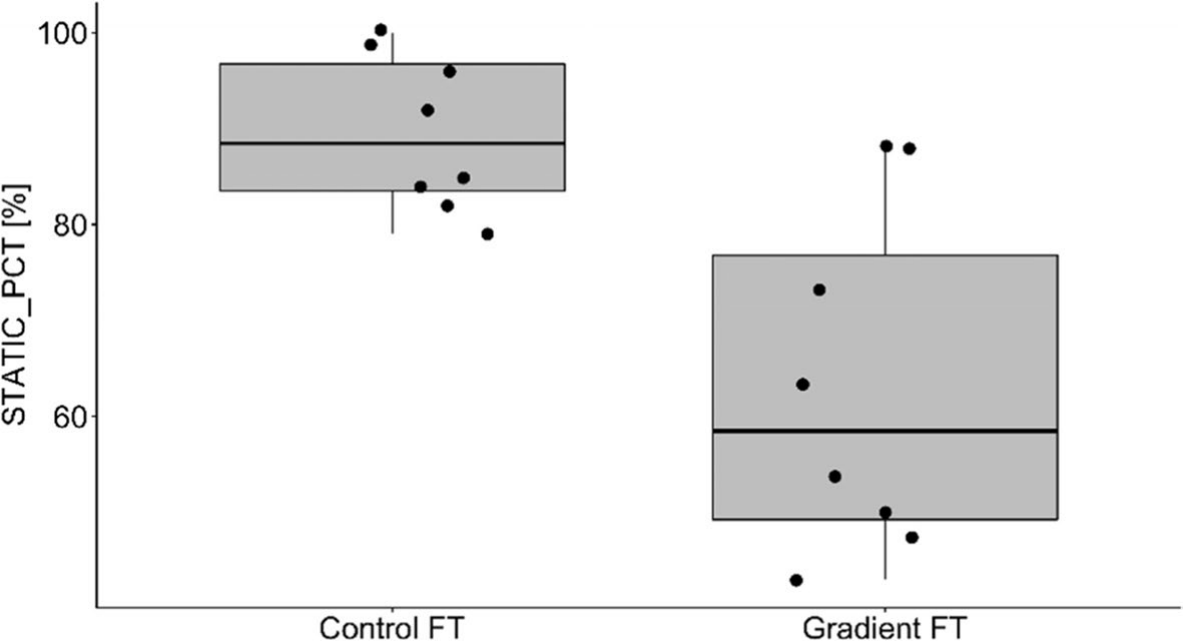
STATIC_PCT- static sperm subpopulations (*p* < 0.05)

The percentage of motile and live spermatozoa was significantly higher in Percoll® group in comparison to the control group (Table 3). There were no differences in proportion of spermatozoa with normal morphology among the groups (Table 3). Samples from the Percolll® group were statistically higher than the control group for straight line velocity (VSL; *p* = 0.015; Fig. 1), Beat Cross Frequency (BCF; *p* = 0.025; Fig. 2), linearity (LIN; *p* = 0.007; Fig. 3) and the percentage of rapid spermatozoa (RAPID_PCT) (*p* = 0.006; Fig. 4). The percentage of static spermatozoa (STATIC_PCT) was significantly higher in the control group (p = 0.007; Fig. 5). Data on functional spermatozoa parameters assessed by fluorescent staining and flow cytometry are presented in Table 4. The populations of cells characterised by an intact acrosome and cell membrane were significantly greater in samples centrifuged in density gradient in comparison with control samples. Percentage of spermatozoa with high mitochondrial potential was higher in the Percoll® group than in the control samples. However, there were no statistically significant differences in proportions of apoptotic cells and spermatozoa with damaged chromatin between both groups (Table 4). The percentage of motile spermatozoa was significantly lower in control group, as compared to the fresh samples. In both control and gradient groups, the percentage of live spermatozoa differed significantly from initial values (Table 3). Compared with the fresh samples, the percentage of motile spermatozoa from cryopreserved samples was significantly lower in both groups.

**Table 3:** The results of microscopic assessment of quality /basic characteristics of wisent epididymal spermatozoa cryopreserved with (Gradient FT) and without (Control FT) pre-freezing Percoll® gradient centrifugation. All data are presented as mean ± s.e. a,b,c, within each column, values with different superscripts are significantly different (*p* < 0.05) with (Gradient FT) and without (Control FT) pre-freezing Percoll® gradient centrifugation

	Motile (%)	Live (%)	Morphologically normal (%)
Fresh	53.75 ± 4.30^a^	84.85 ± 2.12^a^	74.88 ± 3.14^a^
Control FT	26.50 ± 5.24^b^	30.38 ± 4.46^b^	73.63 ± 2.82^a^
Gradient FT	50.00 ± 6.05^a^	53.25 ± 5.39^c^	75.50 ± 3.60^a^

**Table 4:** The functional characteristics of wisent epididymal spermatozoa cryopreserved with (Gradient FT) and without (Control FT) pre-freezing Percoll® gradient centrifugation assessed by flow cytometry. All data are presented as mean ± SEM. a,b,c, within each column, values with different superscripts are significantly different (*p* < 0.05)

	Cells with intact sperm membrane (%)	Cells with intact acrosome (%)	Live non apoptotic cells (%)	Cells with high mitochondrial activity (%)	Cells with damaged chromatin (%)	Cells without lipid peroxidation (%)
24 h from collection	51.46 ± 3.80^a^	53.23 ± 5.55^a^	54.26 ± 9.20^a^	66.37 ± 5.89^a^	0.14 ± 0.05^a^	97.43 ± 0.89^a^
Control FT	11.86 ± 3.60^b^	32.23 ± 4.60^b^	46.26 ± 6.17^a^	20.93 ± 5.78^b^	0.09 ± 0.03^a^	98.15 ± 0.82^a^
Gradient FT	29.93 ± 4.30^c^	40.79 ± 3.80^c^	46.40 ± 2.32^a^	56.94 ± 4.67^c^	0.07 ± 0.04^a^	99.03 ± 0.34^a^

All functional parameters in cryopreserved samples, assessed by flow cytometry, were similar except chromatin integrity which was significantly lower in control group and the viability clearly reduced in both study groups compared to the values in pre-freezing samples (Table 4).

Figures 1, 2, 3, 4 and 5 Characteristic of post- thaw sperm motility assessed by CASA.

## Discussion

Samples containing spermatozoa obtained from wisent epididymis by incision method may be characterised by high content of detritus despite using cell filters [27]. As shown by Martinez-Alborcia et al. (2012) [10], substandard spermatozoa have negative impact on freezability of proper funtioning gametes in boars, as well as, human sperm. Álvarez-Rodríguez (2015) [16] mentioned similar detrimental effects of the presence of blood cells in semen samples. For these reasons, we conducted a study to assess the effectiveness of introduction samples purification stage in the pre-freezing processing of wisent epididymal sperm. Among available selection methods, based on the creation of a physical barrier for reproductive cells [6], we decided to use density gradient centrifugation with a colloidal substance – Percoll®. This substance, formerly popular in human in vitro fertilisation, was excluded from assisted reproductive techniques because of toxic impact on sperm of some batches [15]. Nevertheless we chose this technique due to its widely proven usefulness in artificial breeding in bovine [28–31].

To our knowledge, studies on the use of the Percoll® density gradient centrifugation to select epididymal sperm prior to cryopreservation in animals have not been published. For this reason, it is difficult to compare the results obtained in such procedure. In available literature, there are only a few articles on the use of Percoll® density gradient centrifugation for epididymal sperm [32–35]. Most of the published studies on sperm separation by this method concerning thawed semen [14, 31, 33, 36]. In this research, the use of wisent epididymal sperm selection prior to cryopreservation was described for the first time. A similar procedure was used by Álvarez-Rodríguez M. et al. (2016) in case of the brown bear (*Ursus arctos*). However, in this case, the material was semen obtained by electroejaculation [16].

Such as in cattle, pigs, and brown bear [16] also in this study Percoll® gradient centrifugation allowed to select sperm with high functional properties, which were less sensitive to cryo-damage and thus resulted in higher spermatozoa post thaw quality. In available literature, cited in our bibliography, the use of Percoll® density gradient centrifugation in all cases allowed the selection of samples characterised by higher than initial percentage of motile sperm [28–31, 33, 36]. Such effect was also observed in the case of the wisent epididymal spermatozoa. This process resulted in a statistically significant increase in the post-thawed percentage of motile sperm, in comparison to control group [6]. Percentage of live after thawing spermatozoa was higher in Percoll® separated samples, than in control samples (Table 2). These result was also obtained in case of other ruminants ejaculated semen such as cattle, zebu and red deer epididymal semen [30, 33]. Density gradient centrifugation showed the same capacity while processing the brown bear semen before freezing process as in the case of this study [16]. We observed that higher proportion of spermatozoa with normal morphology was characteristic for samples subjected to density gradient centrifugation (Table 2) which was not found in similar studies of boars [6]. VSL was faster in the Percoll® group than in the control group which was also observed in boars [36]. M. Noguchi et al. (2015) [36] observed similar changes for average path velocity (VAP), curvilinear velocity (VCL), straightness (STR) in boars but these results have not been documented for wisent. M. Noguchi et al. (2015) [36] also noticed that BCF was lower in the boar Percoll® group than in the control group, but comparable results were not detected for wisent in our study. However, in the bull semen samples, as in the case of the wisent, in the group selected with Percoll®, the higher percentage of BCF, LIN and RAPID spermatozoa populations were observed [30].

Populations of cells characterized by an intact acrosome and cell membrane were significantly greater in wisent spermatozoa samples centrifuged in density gradient in comparison to control samples. Comparable results were obtained in boars [36] and bull [15] [31]. L.Z. Oliveira et al. (2012) obtained the same results in the bull intact cell membrane assessment [30]. However, they observed an increase in the percentage of spermatozoa with a damaged acrosome [30]. We observed that the percentage of sperm with high mitochondrial membrane potential was higher in the Percoll® group than in the control samples which was also observed by Arias et al. (2017) [29], Oliveira et al. (2012) in bulls [30]. However, there were no statistically significant differences in the proportions of apoptotic spermatozoa which was also reported for brown bear [16]. There were no differences in proportions of spermatozoa with damaged chromatin between both groups which suggests that centrifugation in Percoll® density gradient has no ability to exclude cells with this defect which was also observed in bull [29].

## Conclusions

In the case of endangered species, each obtained sample is of great importance for the maintenance of the biodiversity of the population. Therefore the main goal of this work was to improve the protocol for handling and cryopreserving wisent epididymal sperm.

This paper describes, for the first time, the use of Percoll® density gradient centrifugation to improve the efficiency of the freezing- thawing process of wisent epididymal sperm. In this study we observed the significant loses from 43.87 to 86.67% in total number of spermatozoa in samples after centrifugation. Despite significant losses, creating a high-quality reserve for in vitro fertilisation purposes, eliminating detritus and improving the overall quality of samples is more important than total number of spermatozoa which justifies the purification of samples by centrifugation in a Percoll® density gradient prior to the cryopreservation process. However, these losses also indicate the need to continue research on methods for the separation of wisent epididymal spermatozoa and further verification in in vitro fertilisation.

## Methods

### Chemicals and media

All reagents and extender components were purchased from Sigma- Aldrich (St. Louis, MO, USA).

### Spermatozoa collection and processing

The material used in this study was obtained and stored pursuant to the permit no. WPN.6401.170.2019.MH issued by the Regional Director for Environmental Protection in Wrocław.

In this study spermatozoa from 4 wisents (*Bison bonasus*, Linnaeus 1758) aged 4–8 years was collected from February to December 2019. Bulls were culled during planned eliminations due to injuries and reproductive disorders under the conditions regulated by Polish law (The Nature Conservation Act 2004). Immediately after animal death, testicles were removed from the scrotum and spermatozoa were obtained by performing multiple incisions in the epididymal tail and immersing the tissue in 4 ml of a sperm-optimized Tris-based extender (Tris (2.4 g), citric acid (1.4 g), glucose (0.8 g), penicillin (5000 IU) streptomycin (100 mg) and distilled water up to 100 ml) (33 °C) placed on glass Petri dishes. Petri dishes were placed on the warming platform for 10 minutes. After incubation, the samples were analysed.

### Initial spermatozoa assessment

The male gametes obtained from each of eight epididymis were processed separately and treated as independent samples in the analyses.

Concentration per unit volume (10^6^ cells/ ml) and motility was assessed using the phase contrast microscope (Nicon Eclipse E200) with warming stage.

To assess the percentage of motile spermatozoa ten microlitres of sample were placed on the slide and covered with a cover slip (× 400).

Concentration in sperm per million (× 10^6^) was counted using Thoma chamber (× 400).

For further morphological assessment, smears from 10 μL of sperm-rich fluid were made and stained with Bydgoska method [37]. To assess the percentage of live and dead spermatozoa, smears from 10 μL of sample and 10 μL of eosin-nigrosin dye were prepared. The following morphological defects where evaluated: proximal droplet, head abnormalities, acrosome abnormalities, midpiece defects, dag-like defect, distal droplet, bent tail, detached head, coiled tail. As a morphological normal sperm were described spermatozoa which did not show these particular defects.

### Preparation of semen

After the initial assessment (concentration and subjective motility) each sample was divided into two parts. One part was diluted with freezing extender I (Tris (2.4 g), citric acid (1.4 g), glucose (0.8 g), egg yolk (20% v/v), penicillin (5000 IU) streptomycin (100 mg) and distilled water up to 100 ml) [38] to obtain the concentration 200 × 10^6^/ml.

The second aliquot was subjected to Percoll® density gradient centrifugation.

### Percoll® density gradient centrifugation

**Fig. 6:**
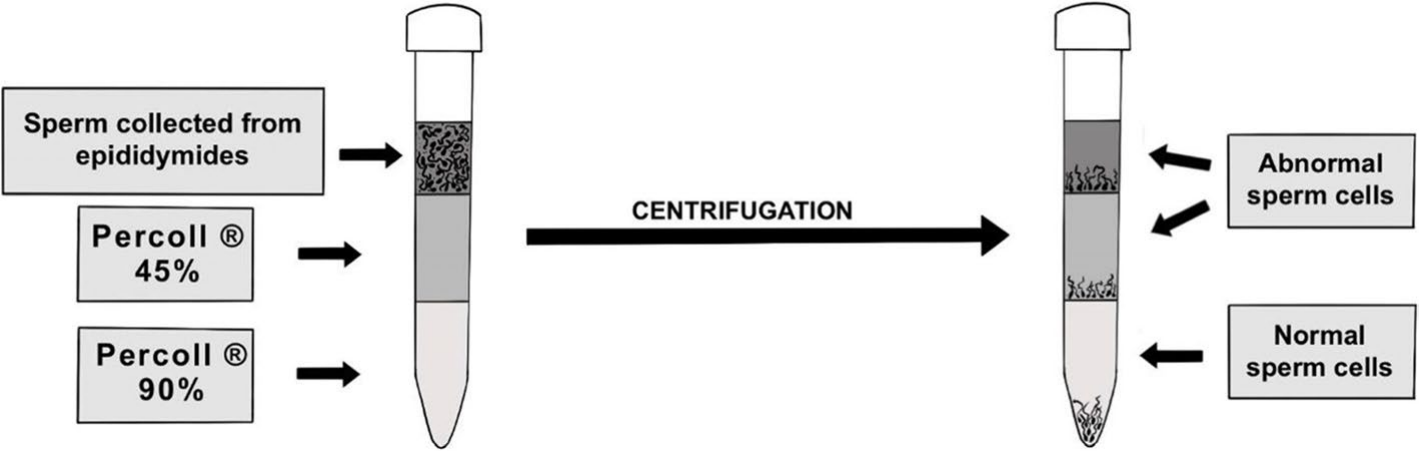
Percoll® density gradient centrifugation

Percoll® solutions (45 and 90%) were prepared as described by Lee et al. (2009) [6] with some modification. Percoll® solution (90%) was prepared by mixing Stock Isotonic Percoll® Solution (SIP) with Human Tubal Fluid (HTF) in a 9:1 proportion. Percoll® solution (45%) was prepared by mixing 90% solution with HTF in equal volume. Subsequently, a 2 ml sample was gently laid on 2 ml 45% Percoll® layer and 2 ml 90% Percoll® layer as presented in Fig. 6. Samples were centrifuged at 800×g for 35 min in a horizontal centrifuge. After removing the supernatant, the pellets were resuspended in 1 ml of HTF and then centrifuged for 5 min at 800×g. After centrifugation, the samples concentrations were diluted with freezing extender I to obtain the concentration 200 × 10^6^ cells/ml.

### Cryopreservation and thawing

The same cryopreservation procedure was used for both group of samples.

After dilution in freezing extender I at 22 °C the samples were kept in a water bath and then placed into a refrigerator and cooled down to 5 °C. Chilled freezing extender II (freezing extender I plus 6% glycerol) was added to obtain final concentration 160 × 10^6^ cells/ml. Diluted spermatozoa were left for further equilibration for 90 min. Subsequently, samples were loaded into 0.25 ml straws (4.0 × 10^6^ spermatozoa per straw). Each free end of the straw was closed with polyvinyl alcohol. Filled straws were frozen in liquid nitrogen vapours (15 min) and then immersed in liquid nitrogen and stored in tanks [38]. From a few weeks to 1 year from the collection day, two straws (one from Percoll® and one from control group) from each epididymis were thawed. The straws were placed in a water bath (37 °C) for 30 sec [38] and subjected to further analysis. To assess the percentage of spermatozoa with morphological defects and the percentage of live and dead gametes, smears were prepared using the same method as described above.

### Assessment of thawed sperm movement parameters with CASA

Motility (MOT, %) and progressive motility (PMOT, %), parameters characterizing sperm movement: VCL (μm/s), VAP (μm/s), VSL (μm/s), LIN (%), STR (%), amplitude of lateral head displacement (ALH) μm), BCF (Hz), Rapid, Medium, Slow and static sperm subpopulations were assessed by using HTM IVOS version 12.2 (Hamilton-Thorne Biosciences Beverly, MA, USA).

The used CASA setups were as for bull spermatozoa: frame rate (60 Hz), frames acquired (30), minimum contrast (80), minimum cell size (5 pixels), low VAP cut-off (30 μm/sec) and low VSL cut-off (15 μm/sec) [38].

### Assessment of the function and structure of spermatozoa by flow cytometry

Spermatozoa were assessed by flow cytometry before and after freezing-thawing process. Due to the long travel distance from the place where the material was obtained to the laboratory where the analyses were performed, the initial pre-freezing assessment was conducted 24 hr. after collection.

Wisent spermatozoa functionality was evaluated using Guava EasyCyte 5 (Merck KGaA, Darmstadt, Germany) cytometer. The fluorescent probes used in the experiment were excited by an argon ion 488 nm laser. Gametes acquisitions were analysed with the GuavaSoft™ 3.1.1 software (Merck KGaA, Darmstadt, Germany). The non-sperm events were gated out based on scatter properties and not analysed. A total of 10,000 events were analysed for each sample. Membrane integrity, acrosome integrity, mitochondrial activity, lipid peroxidation, apoptosis and membrane lipid disorders and chromatin status were assessed [39, 40].

Membrane integrity of wisent spermatozoa was assessed using SYBR-14 stain combined with propidium iodide (PI) (Life Technologies Ltd., Grand Island, NY, USA). 300 μL of sperm- rich fluid was incubated in the dark for 10 min with 5 μL of SYBR-14 working solution (0.1 μL SYBR14 + 4.9 μL TRIS III extender). The analysis was performed after 3 min of incubation with 3 μL of PI. Spermatozoa with intact membranes emit green fluorescence. Cells showing red fluorescence were classified as dead [41].

Acrosome integrity was assessed by lectin PNA stain from *Arachis hypogaea* Alexa Fluor® 488 conjugate (Life Technologies Ltd., Grand Island, NY, USA). Diluted samples were mixed with 10 μL of PNA working solution (1 μg/mL) and incubated for 5 min at room temperature in the dark. Before analysis, the samples were washed and 3 μL of PI was added [42].

Mitochondrial activity was determined using the JC-1 dye (Life Technologies Ltd., Grand Island, NY, USA). 500 μL aliquot of spermatozoa-rich fluid was stained with 0.67 μL JC-1 stock solution (3 mM stock solution of JC-1 in DMSO). The samples were incubated for 20 min at 37 °C in the dark. Spermatozoa emitting orange fluorescence were classified as having high mitochondrial membrane potential (HMMP). Spermatozoa emitting green fluorescence were defined as those with low mitochondrial activity [43].

Lipid peroxidation was evaluated with fluorescent lipid probe C11-BODIPY581/591 (Life Technologies Ltd., Grand Island, NY, USA). One μL of 2 mM C11-BODIPY581/591 in ethanol was added to the diluted sperm- rich fluid and incubated for 30 min at 37 °C in the dark. Subsequently, centrifugation at 500×g for 3 min was performed and the sperm pellets were resuspended in 500 μL of HTF extender. To determine viability, the spermatozoa were stained with PI and incubated for 5 min at room temperature. Spermatozoa which remain unstained were categorised as living population without LPO (L/LPO-) [44].

Apoptosis and membrane lipid disorder were evaluated with YO-PRO-1 dye (25 μM solution in DMSO) (Life Technologies Ltd., Grand Island, NY, USA) (4). 1 μL of YO-PRO-1 stain (final concentration: 25 nM) was added to 1 mL of diluted spermatozoa- rich fluid (500 μL HTF and 500 μL of spermatozoa solution). After incubation for 10 minutes, 3 μL of PI was added before cytometric analysis. Cells showing green fluorescence were classified as YO-PRO-1 positive. Spermatozoa which remain unstained were categorised as living population [44].

Chromatin status was established using the acridine orange dye (AO, Life Technologies Ltd., Grand Island, NY, USA. A spermatozoa-rich solution (100 μL) was subjected to brief acid denaturation by adding 200 μL of the lysis solution (Triton X-100 0.1% (v/v), NaCl 0.15 M, HCl 0.08 M, pH 1.4). After 30 seconds, 600 μL of AO solution (6 μg AO/mL in a buffer: citric acid 0.1 M, Na2HPO4 0.2 M, EDTA 1 mM, NaCl 0.15 M, pH 6) was added. The analysis was performed after 3 minutes of incubation. Spermatozoa with normal DNA configuration were characterised by green fluorescence. Gametes emitting red fluorescence were considered as a population of cells with denatured DNA (DFI) [45].

### Statistical analysis

Statistical analyses were performed by using STATISTICA 13.3 StaSoft (USA). The results of quantitative data are presented as the mean and standard error. Shapiro-Wilk’s test was used to assess data normality. Where appropriate parametric tests (Student’s t-test and ANOVA) or nonparametric tests (Mann-Whitney U and Kruskal–Wallis) were used to evaluate differences between the groups. Differences were considered significant at *p* ≤ 0.05.

## Data Availability

The datasets used during the current study are available from the corresponding author on reasonable request.

## Abbreviations

ALH: Amplitude of lateral head displacement
AO: Acridine orange dye
ART: Assisted reproductive techniques
BCF: Beat cross frequency
CASA: Computer-assisted semen analysis
HMMP: High mitochondrial membrane potential
HTF: Human tubal fluid
LIN: Linearity
MOT: Motility
PI: Propidium iodide
PMOT: Progressive motility
PVP: Polyvinylpyrrolidone
RAPID, RAPID_PCT: The percentage of rapid spermatozoa
ROS: Reactive oxygen species
SIP: Stock Isotonic Percoll® Solution
STATIC_PCT: Percentage of static spermatozoa
STR: Straightness
VAP: Average path velocity
VCL: Curvilinear velocity
VSL: Straight line velocity
